# The Impact of Social Media on Health Behaviors, a Systematic Review

**DOI:** 10.3390/healthcare13212763

**Published:** 2025-10-30

**Authors:** Bernadette Paul, Sely-Ann Headley-Johnson

**Affiliations:** 1 School of Applied Human Development, College of Education & Human Development, Bowling Green State University, Bowling Green, OH 43403, USA; 2 Health Sciences Department, Purdue Global University, West Lafayette, IN 47907, USA; selyann.headleyjohnson@purdueglobal.edu

**Keywords:** health behavior, social media, mental health, diet, physical activity, body image, substance use

## Abstract

**Background**: Social media has transformed and influenced how health information is accessed, how people engage in health-promoting behaviors, and how they develop attitudes toward wellness. **Methods**: This systematic review examines the impact of social media on various health behaviors from 2010 to 2025. Guided by the PRISMA-ScR framework, and after screening 436 articles from databases such as PubMed, PsycINFO, Scopus, and CINAHL, 82 studies were included that were published in English, and examined Social Media’s influence on health-related behaviors. **Results**: Thematic synthesis revealed five dominant categories: physical activity and fitness, dietary behaviors and nutrition, mental health and wellbeing, substance use and risky behavior, and health misinformation. The platform, content exposure, and user engagement are all factors that can contribute to social media being an enabler and an obstacle to positive health behavior change. **Conclusions**: This review identifies key knowledge gaps, highlights emerging trends, and provides direction for future interdisciplinary research and public health strategies. This review was not registered and received no funding.

## 1. Introduction

Social media is a general term used to describe different online platforms such as blogs, business networks, collaborative projects, enterprise social networks (SN), forums, microblogs, photo sharing, products reviews, social bookmarking, social gaming, video sharing, and virtual worlds [1]. Over the past years, social media platforms such as Facebook, Twitter/X, Instagram, Snapchat, and TikTok have become important components of daily life, affecting how people access, share, and internalize health-related information. With more than 5 billion users globally, social media’s influence on health behaviors spans mental wellness, physical activity, diet, body image, and substance use [2,3].

Social media shows potential for being a platform for health promotion, but it also carries the risk of disseminating misinformation, exacerbating health disparities, and fostering maladaptive behaviors such as disordered eating, substance misuse, or vaccine hesitancy [4,5,6]. Understanding how social media influences health behaviors is fundamental for designing effective interventions, guiding public health policy, and improving health outcomes in an increasingly digital society.

While correlational evidence links high social media use to depression, anxiety, disordered eating, and poor sleep among youth [7,8,9] platforms also offer unprecedented opportunities for health promotion, support groups, and behavior-change campaigns [3,10]. While other reviews focus on particular segments of public health, this review aims to map and synthesize evidence between 2010 and 2025 on how social media impacts general health behaviors, while identifying knowledge gaps, and advise future research and policy.

### 1.1. Defining Health Behaviors in the Digital Age

Health behavior refers to any reactive, intentional, or habitual behavior by an individual that could positively or negatively affect their physical and mental health [11]. Another definition describes health behaviors as actions that influence their health status [12]. These actions include exercise routines, dietary choices, substance use, and stress management, and are shaped by a constant flow of content, peer influence, algorithmic personalization, and social comparison [13]. Emerging evidence suggests that social media not only informs but also transforms health behavior intentions and outcomes. Whether through weight loss challenges on TikTok, mindfulness campaigns on YouTube, or harm-reduction messaging on Reddit, social media has become a powerful yet unpredictable force in health decision-making [14,15].

### 1.2. Rationale for the Review

A review allows exploration of the nature, scope and extent of the multidisciplinary and complex literature [16,17]. A preliminary search of various search engines revealed that no reviews on the topic were identified. Furthermore, existing reviews focus on specific behaviors or populations, for example, social media’s impact on smoking cessation and on mental health. Hence, a comprehensive literature review is needed to understand the impact of social media on health behavior.

### 1.3. Objectives

The main objective of this review is to explore the influence social media has on health behaviors across various populations and domains from 2010 to 2025. This review aims to specifically:

- Identify the most frequently studied health behaviors that are influenced by social media.
- Explore theoretical frameworks that are used to understand these influences.
- Examine population subgroups that are most affected.
- Identify knowledge gaps and provide suggestions for future research.

### 1.4. Research Questions

1. What health behaviors are most influenced by social media use?
2. How do different platforms such as Instagram, TikTok, Twitter/X, and Facebook impact specific health behaviors?
3. What theoretical models explain the link between the use of social media and behavior change?
4. What populations are most studied in this field?

### 1.5. Significance of the Review

This review will contribute to understanding social media’s role in reshaping public health landscapes. The evidence will inform researchers, policymakers, and practitioners on what should be done to regulate health misinformation, how to leverage platforms to promote sustainable health behavior change, and how to design targeted interventions.

## 2. Materials and Methods

Social media’s impact on health behaviors was explored using a systematic review that was guided by the methodologies of the Cochrane handbook for systematic reviews and aligned with the Preferred Reporting Items for Systematic Reviews and Meta-analyses extension for Systematic Reviews (PRISMA) [16,17,18]. This approach is appropriate for the heterogeneous and rapidly evolving body of literature examining the impact of social media on health behaviors.

### 2.1. Inclusion and Exclusion Criteria

The inclusion and exclusion criteria were developed using the PCC (Population, Concept, Context) framework [19].

Population: Individuals of any age, background, or gender who use social media platforms.

Concept: The influence of social media on any health-related behavior, such as diet, mental health, substance use, use of health services, and physical activity.

Context: Studies conducted in any geographical location and published in English between 2010 and 2025.

### 2.2. Inclusion Criteria

Empirical studies (quantitative, qualitative, or mixed methods).

Peer-reviewed articles, conference proceedings, and grey literature.

Studies exploring any mainstream or niche social media platforms such as Facebook, Instagram, TikTok, Twitter/X, Reddit, YouTube.

Articles investigating behavioral outcomes such as exercise frequency, smoking cessation, dietary changes, and mental health self-management.

### 2.3. Exclusion Criteria

Editorials, commentaries, or opinion pieces without empirical data.

Studies focusing solely on traditional media (e.g., television, radio).

Articles not published in English.

Studies without a clear focus on behavior change or health outcomes.

### 2.4. Information Sources and Search Strategy

Keywords included “social media,” “health behavior,” “mental health,” “diet,” “physical activity,” “body image,” “substance use,” and related terms. Gray literature from government and NGO sources (e.g., Surgeon General, APA) was also included. Exclusionary language to remove irrelevant fields was also applied. For example: NOT (advertising OR “social marketing” OR “business” OR “e-commerce” OR “algorithm” OR “computer science”). Searches were conducted between January and June 2025 and included the following databases:

- CINHAL
- ERIC
- Google Scholar
- PubMed
- PsycINFO
- ProQuest
- Scopus
- Sage
- Web of Science
- Wiley

### 2.5. Study Selection Process

A total of 412 articles were initially retrieved from database searches. After removing 306 duplicates, 130 unique studies remained for title and abstract screening. Of these, 12 articles were excluded for not meeting the inclusion criteria. Full-text retrieval was attempted for the remaining 118 articles; however, three could not be retrieved. Of these 115, an additional 33 reports were excluded due to incorrect research period, non-English articles, and inability to access the full article. After the entire screening process, 82 eligible articles were used to complete the final review. The selection process is illustrated in the PRISMA-ScR flow diagram (Appendix A). The supporting information in File S1: The PRISMA 2020 checklist can be downloaded at https://www.prisma-statement.org/prisma-2020-checklist.

For the thematic analysis, data from the included articles were coded using a highlighting and grouping approach. An indictive coding and thematic approach was applied, allowing themes to emerge from the articles rather than from predetermined categories. Recurring patterns and recurring concepts were then grouped into themes. This process involved systematically reviewing the included studies to identify relevant text. The relevant text was reviewed for recurring ideas and patterns and then grouped into broader themes. Only one author conducted the coding and thematic grouping, which ensured consistency but may have introduced interpretation bias. Additionally, a narrative assessment was also done, considering each study’s methodological clarity, sample characteristics, and potential sources of bias.

## 3. Results

Using the PRISMA process, 82 articles including varying designs that spanned a global range of contexts and populations were included in the final synthesis. The results are categorized into the following key themes:

### 3.1. Social Media and Mental Health Behaviors

- Protective nuance: Social media platforms such as Reddit, Instagram, and YouTube have served as mediators to mental health advocacy, peer support groups, and coping strategies for individuals with serious mental health issues [20]
- Correlation with distress: While social media can serve as a form of social support, exposure to harmful content can negatively influence vulnerable users [21,22]. Frequent and problematic social media use correlates with depression, anxiety, lower self-esteem, self-harm, and suicidality in youth [8,23]. Passive use of social media such as scrolling, was shown to be more harmful than active use [24,25].
- Anxiety and Fear of Missing Out (FOMO): Studies have shown that the more time spent on Instagram and Snapchat has been correlated with FOMO, and symptoms of anxiety, especially in adolescents [26]. Furthermore, increased social comparison and depressive symptoms were associated with scrolling (passive consumption) instead of posting (active engagement) [25]
- Therapeutic Interventions and Mental Health Campaigns: Mental health campaigns like “#BellLetsTalk, and #MentalHealthAwarenessMonth” that are carried out on social media show potential in reducing stigma and increasing help-seeking behaviors [27].

### 3.2. Nutrition, Diet & Obesity

Social media is a source of accurate and inaccurate nutrition information and has been known to positively and negatively influence dietary habits and trends. Exposure to diet culture on Instagram significantly predicted restrained eating, body dissatisfaction, and use of appetite suppressants [13]. TikTok’s “What I Eat in a Day” trend was flagged as a driver of social comparison and caloric restriction among teens [28].

Several studies suggest that social media can positively influence dietary behavior changes when evidence-based messages are shared by qualified professionals. Positive influences included increased awareness of plant-based diets, healthy recipes, and calorie tracking apps promoted by influencers [12,29]. Dietitians who used Instagram to post evidence-backed recipes and tips saw higher engagement and follower dietary improvements [30,31].

Lots of materials such as detox videos, keto meal plans, vegan recipes, and “what I eat in a day” videos have flooded YouTube, TikTok and Instagram [13,32], with little to no mention of potentially negative effects and people that should not partake in these activities.

### 3.3. Body Image & Cosmetic Behaviors

Social media has provided an ample platform for body dysmorphia disorder (BDD) [33]. Image-based platforms like Instagram have been negatively associated with disordered eating behaviors and body image. Instagram has been associated with orthorexia and binge eating disorder [3,13]

### 3.4. Social Media as Motivational and Engagement Tools

Social media platforms combined with goal setting, self-monitoring, and peer accountability serve as effective motivating tools to help individuals engage in physical activity. Facebook groups, Instagram fitness challenges, and Twitter/X campaigns have been associated with increased physical activity in adults and adolescents [34,35]. Platforms such as Instagram often promote “fitspiration” content, and visual and textual encouragement for fitness, which has been linked to increased exercise intention [36]. It must be noted that the impact of this content is complex. While it can be inspiring, it can also induce appearance-related anxiety [37].

### 3.5. Wearable Tech Integration and Gamification

Fitbit and Strava are social media-integrated wearable technologies, and they enhance physical activity through gamification features like leaderboard competitions and milestone sharing [38,39]. These elements exploit social comparison to promote sustained behavior change.

### 3.6. Population-Specific Outcomes

#### 3.6.1. Youth and Adolescents

Social media-based interventions when delivered via platforms like TikTok and Snapchat, which are frequently used by adolescents, were more effective [40,41].

#### 3.6.2. Older Adults

Digital literacy was a deterrent, but with training, older adults showed increased walking and reduced sedentary behavior using Facebook-based support groups [42].

### 3.7. Social Media and Normalization of Risky Behaviors

Numerous studies have found that social media content can normalize or glamorize risky health behaviors, particularly substance use. Platforms such as Instagram, TikTok, and Snapchat frequently feature alcohol, marijuana, vaping, and party-related content often shared without health warnings by influencers or peer networks [21,43].

TikTok videos tagged with hashtags like #DrunkTok or #420 frequently portray substance use in a humorous or appealing way. Rutherford and colleagues found that more than 70% of marijuana-related content on TikTok positively portrayed cannabis use, often by young creators [44].

### 3.8. Peer Influence and Behavioral Modeling

Exposure to peer-generated content involving drinking or drug use increases the likelihood of imitative behavior among adolescents [45]. The social reward mechanisms (likes, shares, comments) reinforce such behaviors and create perceived norms of acceptability. Research shows that alcohol use can be related to frequent exposure to alcohol-related content on social media [46].

### 3.9. Counter-Narratives and Harm Reduction

Despite the many risks, some social media initiatives have effectively promoted harm reduction. Communities hosted by social media platforms provide resources on safe substance use, overdose prevention, and pathways to recovery [47].

For example, Reddit’s “r/StopDrinking” and “r/leaves” (for quitting cannabis) offer anonymity and peer support, demonstrating measurable positive outcomes in substance reduction and relapse prevention [48].

### 3.10. Social Media and the Reach of Health Misinformation

Social media is a major facilitator for the spread of health misinformation, especially during public health crises. Misinformation was widely proliferated on Facebook, YouTube, and Twitter/X regarding COVID-19 vaccines, cancer cures, and unproven diet fads [49,50].

Emotional appeal and sensationalism are two reasons why misinformation spreads faster and more broadly than factual information [51,52]. False claims such as “alkaline diets cure cancer” or “vaccines cause infertility” can dissuade individuals from seeking appropriate care.

### 3.11. Influencer Impact and Conflicts of Interest

Health influencers often lack formal training but wield substantial sway. Digital influencers are perceived as relevant because of the shared experiences and opinions they provide [53]. Sponsored content, such as detox teas, and miracle supplements are commonly promoted by fitness or wellness influencers with limited evidence-based backing [54].

A small number of influencers disclose conflicts of interest or cite scientific sources, which contributes to misinformation and commercialization of health behaviors [52,55].

### 3.12. Response and Misinformation Mitigation

Content moderation strategies such as misinformation warnings, algorithm demotion, and promotion of authoritative sources have been implemented by social media platforms. Partnerships between health authorities and social media platforms have allowed the display of information panels on vaccine content [56].

However, these measures are inconsistently applied and often delayed. Many users continue to engage with misinformation based on their beliefs [51].

## 4. Discussion

The results of this review underscore the profound and multifaceted impact of social media on health behaviors across diverse populations between 2010 and 2025. Social media platforms have created unique opportunities for health promotion, peer support, and access to health information, and have simultaneously introduced significant risks related to mental health, body image, substance use, and misinformation.

### 4.1. Dual Nature of Social Media’s Influence

This review revealed a very significant theme, which is the dual nature of social media’s impact. We found that platforms such as Instagram, Facebook, TikTok, and YouTube have aided health education, peer support, and self-management behaviors through community-driven groups, health influencers, and digital health interventions [3,57]. For example, patients living with chronic conditions such as diabetes or depression have used social media to share their lived experiences and to receive emotional support, which often results in improved self-efficacy and health literacy [58,59].

On the other hand, the negative behavioral and psychological consequences, especially among adolescents and young adults have become increasingly apparent. Excessive social media use has been associated with poor sleep hygiene, reduced physical activity, increased sedentary time, poor dietary habits, and elevated risks of depression, anxiety, self-harm, and disordered eating behaviors [8,9,60]. This duality highlights the need for targeted interventions and digital health literacy education, especially among younger users.

### 4.2. Mental Health Outcomes: The Growing Crisis

Many studies in this review have linked problematic or excessive social media use with poor mental health outcomes. Adolescents, in particular, are vulnerable to the curated and often idealized portrayals of life online, which can lead to negative social comparisons, cyberbullying, and feelings of inadequacy [60]. A growing body of evidence demonstrates that platforms promoting appearance-based content, such as Instagram and TikTok, exacerbate symptoms of anxiety and depression, particularly among females [36,61].

However, some studies have indicated moderate or context-specific benefits such as social connection, emotional expression, and community belonging [62]. The subtle differences in these views support public health messaging that emphasizes healthy engagement, rather than abstinence, and advocates for design changes to reduce addictive features [63].

### 4.3. Physical Activity, Diet, and Obesity

This review revealed that increased screen time and prolonged social media use correlate with sedentary behavior and decreased physical activity, particularly among youth [64]. Social media often exposes users to unhealthy dietary trends, such as detox teas, extreme calorie restrictions, or unregulated weight loss supplements [13,32]. The influence of “fitspiration” content is complex, and it may encourage exercise in some but promote unrealistic body ideals and compulsive exercise in others [3,65]. Furthermore, TikTok and Instagram algorithms that promote dietary restrictions, fasting trends, or “what I eat in a day” videos have been connected to disordered eating and body dissatisfaction [66,67]. These findings highlight the need for and the importance of content regulation, algorithm transparency, and inclusion of diverse, evidence-based health messaging.

### 4.4. Body Image, Cosmetic Behavior, and Dysmorphia

The popularity of the use of filters, edited images, and content focusing on appearance has contributed to what has been described as “Snapchat dysmorphia”. “Snapchat dysmorphia” is a phenomenon where individuals seek cosmetic procedures that make them resemble their filtered digital selves [68]. “Snapchat dysmorphia” highlights how frequent exposure to idealized or altered images can distort users’ perceptions of beauty, and may trigger body dysmorphic disorder and low self-esteem, especially in adolescents and young women [69,70].

Several longitudinal studies included in this review confirm that high engagement with appearance-based content predicts stronger internalization of thin or muscular ideals, and increased interest in plastic surgery or aesthetic modification [21,70,71]. High engagement with appearance-based content appears to be more extreme among users who compare themselves to influencers or peers with large followings, suggesting the need for training in media literacy and promotion of positive body image in educational curricula.

### 4.5. Risk Behaviors and Substance Use

Social media has been associated with normalizing risky behaviors, such as alcohol consumption, vaping, and drug use. Social media platforms that use viral “challenges” or influencer endorsements to showcase or glamorize substance use have been linked to teenagers increased intentions and actual use of alcohol and nicotine [21,72]. TikTok is one of the platforms that has drawn criticism for its relaxed moderation of such content [73]. Well-designed targeted public health campaigns have shown potential in reducing risky behavior and increasing awareness [74]. It must be noted that while social media may contribute to the problem, it can also be a powerful vehicle for harm reduction if it is purposefully used.

### 4.6. Health Communication, Campaigns, and Misinformation

Social media has positively and negatively impacted Health communication. The swift dissemination of information during public health crises such as COVID-19, enabling real-time updates and behavior change interventions [75]. However, when used as a source for misinformation, social media can damage public trust and lead to harmful health decisions, conspiracy theories, and vaccine hesitancy [4].

This review identified that health campaigns on platforms such as YouTube, Twitter/X, and TikTok are most effective when they involve peer-to-peer messaging, user-generated content, and cultural tailoring [76,77]. However, key challenges such as algorithmic opacity and the viral nature of misinformation remain.

### 4.7. Moderators and Vulnerable Populations

Age, gender, socioeconomic status, mental health status, and offline support networks some of the factors that impact the relationship between the use of social media and health behaviors. [78,79]. Youth with strong offline social support may experience less harm while those with pre-existing anxiety or depression seem to be more vulnerable to the negative effects of social media [24]. Social media is often used as a space for community and identity formation by groups such as LGBTQ+ youth, racial minorities, and individuals with chronic illnesses [80,81,82]. For these groups, online support may be both empowering and protective against stigma.

## 5. Research Gaps and Future Directions

In spite of the rapid expansion of research on social media and health behaviors, this review identified several persistent gaps in theory, evidence, and methodology. These gaps limit our understanding of causal mechanisms, population-level impacts, and the development of effective interventions.

### 5.1. Platform-Specific Research Imbalance

Facebook and Instagram were the most studied platforms. However, TikTok, Reddit, and newer platforms like Discord or Threads—which are increasingly popular, especially among youth, remain underexplored.

Future Direction: Future research should investigate less-studied and emerging platforms to understand their unique features, user engagement styles, and influence on health behaviors. Each platform has distinct characteristics, such as anonymity, short-form video, and community norms, which require tailored analytical approaches.

### 5.2. Misinformation Mitigation and Moderation Gaps

Although misinformation emerged as a major theme, few studies evaluated the effectiveness of countermeasures such as content labeling, influencer disclaimers, health literacy education, and algorithmic filtering.

Future Direction: Rigorous studies are needed to test the efficacy of misinformation interventions. Using mixed-method and quasi-experimental studies that simulate real-world information exposure could help evaluate how users respond to fact-checking, disclaimers, or authoritative content nudges.

### 5.3. Insufficient Focus on Digital Health Equity

More studies are needed to address digital disparities among marginalized populations that include racial and ethnic minorities and LGBTQ+ communities, individuals with low literacy or rural access, and older adults with low digital fluency

Future Direction: Researchers should adopt an equity lens and engage underrepresented communities using participatory design methods. Intervention studies should stratify results by demographic variables to evaluate differential impacts and accessibility.

### 5.4. Disordered Eating and Social Comparison

Although the link between social media and body image concerns is well-documented, research often fails to distinguish between healthy motivation (e.g., fitspiration) and pathological comparison (e.g., thinspiration, orthorexia).

Future Direction: Thorough research is needed to evaluate the different dimensions of disordered eating and social media, exploring psychological mediators such as self-esteem, perfectionism, and emotional regulation. Experimental designs using manipulated content exposure could shed light on causal pathways.

### 5.5. Community and Peer Support Mechanisms

Online peer support emerged as a motivator in physical activity, substance cessation, and mental health. However, the dynamics and efficacy of digital peer support are not well understood.

Future Direction: Future studies should explore the quality, depth, and type of peer interactions that lead to sustained behavior change. Social network analysis could identify which user roles (e.g., moderators, influencers, community leaders) are most impactful.

### 5.6. Integration with Health Systems and Providers

Few studies evaluated how social media interventions could be integrated with formal healthcare systems. Most health campaigns on social media remain disconnected from clinical care, limiting continuity and accountability.

Future Direction: Implementation of science-based approaches should be applied to examine how health systems, public health agencies, and providers can incorporate social media-based behavior change tools into patient education and chronic disease management.

### 5.7. Ethical and Regulatory Research

Ethical questions related to privacy, data use, content moderation, and psychological harm are rarely addressed, and the control of health-related content by tech companies remains unclear.

Future Direction: Policy-oriented research should evaluate the ethics of platform-based health interventions, including the trade-offs between algorithmic targeting and user autonomy. Researchers should advocate for ethical data use standards in digital health research.

## 6. Limitations

This study has a few limitations. Firstly, only one author conducted the coding and thematic analysis, which may have introduced selection bias and subjectivity in theme identification and interpretation. Future reviews could strengthen reliability by involving multiple coders and using interrater agreement to validate the coding framework.

Secondly, the study examined general impacts of social media on overall health behavior rather than focusing on a specific domain, which may limit the depth of the findings. Thirdly, the 15-year study period may not be sufficient to capture long-term shifts or more recent rapid changes in social media use. Additionally, the review included only English-language articles, which may exclude relevant findings from non-English research. Moreover, the measures of social media exposure and health behavior outcomes were often self-reported, introducing recall and reporting bias. Also, many studies focused on younger populations, reducing the generalizability of these findings.

## 7. Conclusions

Evidence has shown that between 2010 and 2025, social media has evolved into a powerful and often decisive context for health behavior. This review shows an apparent dichotomy: the same platforms that amplify health education, peer connection, and self-management can also increase risks tied to mental health, body image, disordered eating, sedentary behavior, substance use, and misinformation. The task ahead is not to choose between celebrating or condemning social media, but rather to intentionally manage its risk–benefit profile.

The evidence suggests three reinforcing options for harm reduction and the amplification of benefits. First, build digital health literacy across lifespans by teaching users to evaluate sources, recognize manipulative designs, and practice healthier engagement strategies (e.g., setting time boundaries, diversifying feeds, and engaging in critical comparison). Second, design for health: default safety settings for youth; friction for potentially harmful features (endless scroll, algorithmic rabbit holes); transparent labeling of promotional or appearance-altered content; and easy access to credible, culturally tailored information. Third, embed social media within care and public health by linking digital peer support and campaigns to clinical pathways, school and community programming, and crisis resources for timely, traceable, and equitable help.

Mental health findings, especially among adolescents and young adults, need coordinated responses. Clinicians and school health professionals can routinely screen for problematic use, sleep disruption, and body image concerns; counsel on healthy digital routines; and refer to evidence-based supports. Families and educators can cultivate protective offline networks and media-literacy curricula that reduce harmful social comparison while preserving the benefits of community and self-expression. Public health agencies can partner with credible creators to seed engaging, peer-led content that competes with low-quality narratives without moralizing or stigma.

Physical activity, diet, and body image outcomes highlight the need for accountability in content and algorithms. Platforms should be encouraged and guided on curbing the amplification of extreme dieting, unregulated supplements, and appearance-distorting content, while elevating diverse body representations and realistic health behaviors. “Fitspiration” can be redirected toward competence, enjoyment, and function—not aesthetics—by emphasizing inclusive role models and attainable goals.

Risk behaviors and substance use highlight that the medium can carry either promotion or prevention. When public health campaigns mirror the aesthetic, pacing, and participatory norms of each platform—and leverage community leaders and micro-influencers—they can shift norms, challenge glamorization, and support cessation. Coordination with schools, youth organizations, and healthcare increases reach and sustainment.

Equity must anchor all efforts. Marginalized groups benefit from the community and identity affirmation social media can provide, yet face disproportionate harms from stigma, targeted misinformation, and access barriers. Co-design with affected communities, accessibility by default (low literacy, low bandwidth), and routine equity stratification in evaluations are essential to ensure solutions do not widen gaps.

Methodologically, the field should move beyond cross-sectional snapshots toward longitudinal, experimental, and implementation studies that reflect real-world exposure and platform evolution. Partnerships that protect privacy while enabling independent auditing of algorithms and interventions will be critical. Clear ethical guardrails for data use, transparency, and psychological safety should accompany any scale-up.

In summary, social media is neither a panacea nor a pathology; it is an environment that we can shape. A pragmatic strategy that improves literacy, reforms design, integrates care and public health, and centers on equity—can preserve connections, learning, and empowerment while reducing preventable harm. With collaborative and coordinated action from researchers, clinicians, educators, public health practitioners, platform designers, and policymakers, social media can be designed to meet higher standards, creating a healthier and more trustworthy digital ecosystem.

## Figures and Tables

**Figure A1:**
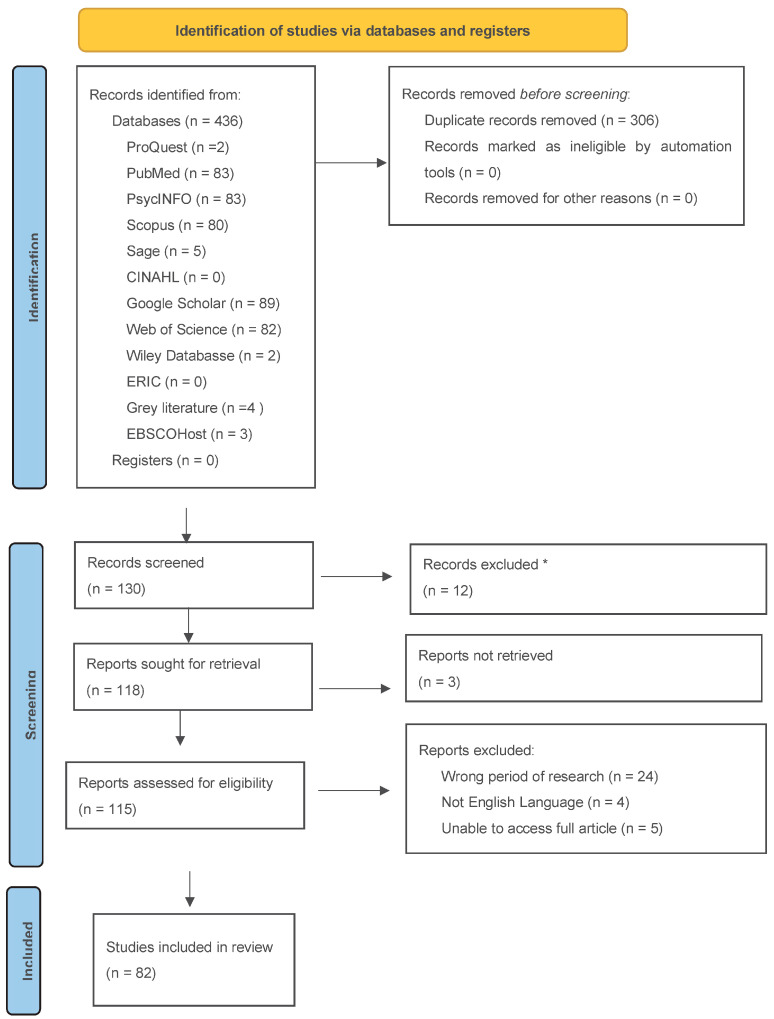
PRISMA Flow Diagram. * With the exception of the search engines, no automation tools were used. We manually excluded articles based on exclusion criteria.

## Data Availability

No new data were created or analyzed in this study. Data sharing is not applicable to this article.

## Supplementary Materials

The following supporting information can be downloaded at: https://www.mdpi.com/article/10.3390/healthcare13212763/s1, File S1: The PRISMA 2020 checklist.
